# Recognition and Management of Oculogyric Crisis in a Learning Disability Setting

**DOI:** 10.1192/bjo.2026.11880

**Published:** 2026-06-30

**Authors:** Ritesh Ramachandra, Jennifer Dolman

**Affiliations:** Hampshire and Isle of Wight Healthcare NHS Foundation Trust, Southampton, United Kingdom

## Abstract

**Aims:**

Oculogyric Crisis (OCG) is one of the few presentations within psychiatry considered an emergency due to the potential features which can make OCG life-threatening. OCG is poorly understood and can be difficult to recognise due to other features which may mask the classical presentation of a fixed upper gaze of the eyes with associated anxiety and hallucinations. This case study looks at how we have managed a patient within a low secure forensic service with this presentation.

**Methods:**

Our male patient in their 30’s was admitted with a working diagnosis of treatment resistant schizophrenia. There were frequent episodes of this gentleman becoming acutely unwell with vivid hallucinations and aggression directed towards staff and other patients. Due to staffing concerns regarding autonomic function, the associated eye movements were missed and as such this was not recognised as OCG which is an acute dystonic reaction.

**Results:**

During these episodes, there were many times where our patient would appear in an immobile state, with an inability to speak or follow commands with marked feelings of reference and ideas of persecution. This would include refusals to take prescribed medication because of visual hallucinations of seeing his medication and food and drink being contaminated. Episodes of seclusion were due to aggression towards others he believed were after him. Frequent emergency department attendances were required due to autonomic and conscious level disturbance.

Our patient was admitted on a regular depot of zuclopenthixol which we gradually reduced to nothing following the recognition of OCG. We also supplemented this with procyclidine, which helped to reduce symptoms.

Following these changes, we initially noticed a marked improvement in our patient’s presentation as he was engaging well with the ward staff and other patients. There was no longer any requirement for frequent hospital visits and the absence of a movement disorder.

However, they appear to have escalated again following complete cessation of the depot. We need to understand if this is an exacerbation of OGC, which the literature does document when antipsychotic medication is withdrawn or an exacerbation of the schizophrenia due to reduction in antipsychotic medication, even though he remains on an oral atypical antipsychotic known to have minimal risk for side effects.

**Conclusion:**

OCG is a rare but serious condition. However, if it is recognised early, an individualised approach based on the patient and clinical picture may improve long term outcomes whilst considering alternative diagnoses.

